# How the social lives of bacteria affect their pangenome

**DOI:** 10.1042/EBC20250039

**Published:** 2026-09-02

**Authors:** Fiona Jane Whelan

**Affiliations:** School of Biological Sciences, Faculty of Biology Medicine and Health, University of Manchester, Manchester, U.K.

**Keywords:** bioinformatics, meta-pangenomes, metagenomics, microbe-microbe interactions, microbiome, pangenomes

## Abstract

Although the study of microbes started with type strains and reference genomes, advances in sequencing technology and new interest in mixed microbial communities have made us aware that a single genome cannot and does not reflect the diversity of a given bacterial species. Bacteria rarely occupy an environmental or host niche alone and quickly diversify into strains upon colonization of a new niche. The genetic diversity present within a phylogenetically related set of bacterial strains (the ‘pangenome’) is influenced by the niche that they occupy and how they interact with the other microorganisms that they share that niche with. In this review, I examine how the social lives of bacteria can affect their genetic diversity and the bioinformatic techniques that we use to detect that diversity.

## Defining the microbial pangenome

**Figure 1:**
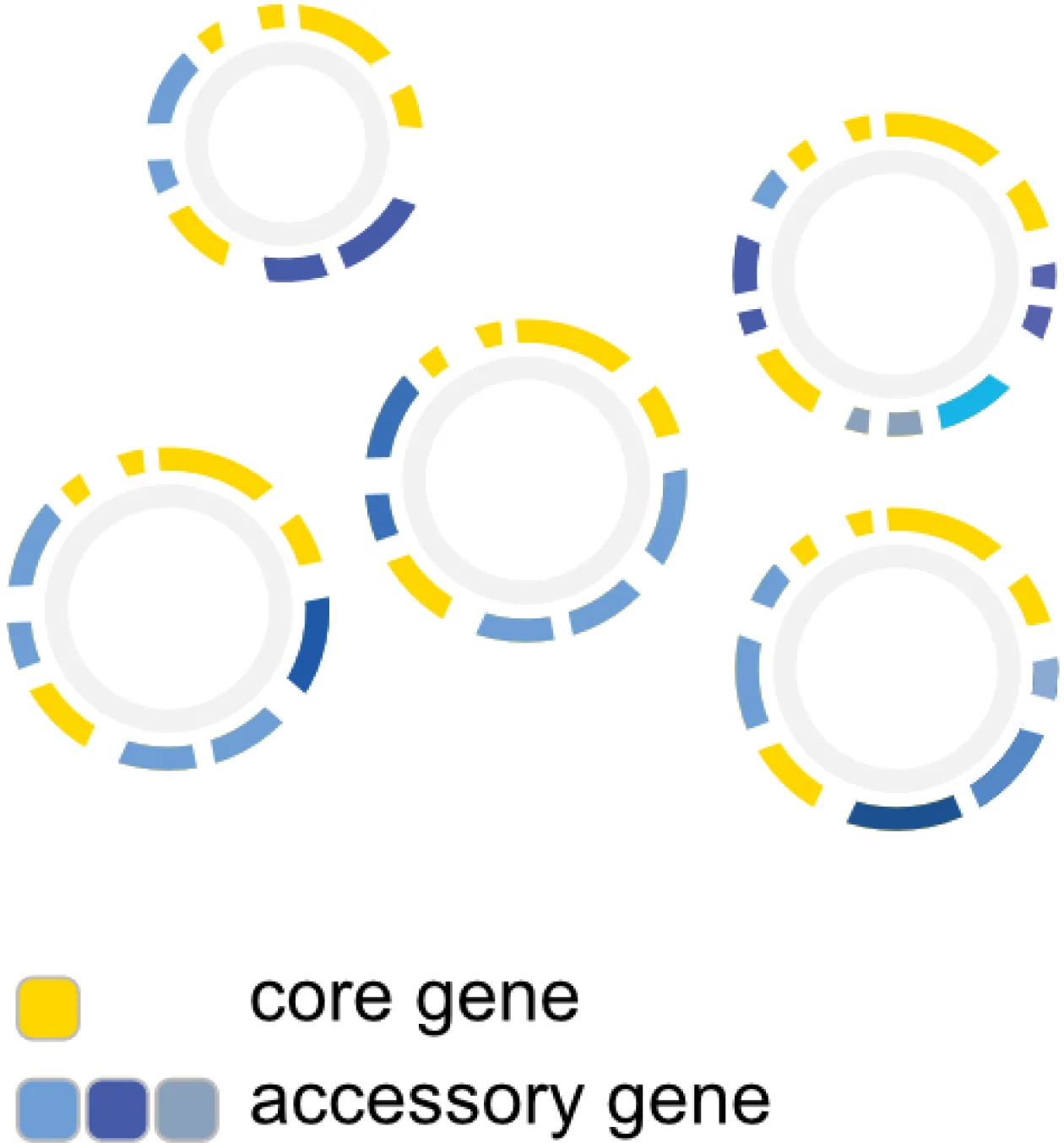
A cartoon depiction of a microbial pangenome Within a pangenome, core genes (**yellow**) are consistent across genomes whereas accessory genes (blue) are only found in a subset of genomes.

A pangenome is defined as the totality of the genetic content of all strains of a bacterial population (usually defined at the level of a species) [1,2]. This genetic diversity can be split into genes that are core—present in all—and accessory—present in some strains (Figure 1). The term ‘pangenome’ was coined as part of an analysis of 8 genomes of *Streptococcus salivarius* [1]; in the 20 years since, pangenome analyses have expanded to studies including, for example, 12,560 *Escherichia coli* [3] and 3,052 *Streptococcus spp*. genomes [4]. Large-scale investigations of prokaryote pangenomes have largely been made possible due to improvements in DNA sequencing technologies; however, the development and continued improvement of bioinformatic tools to analyze large bacterial datasets has also been vital for this success. Early tools, such as Roary [5] and PanGP [6], laid the groundwork for improvements to speed, throughput, and/or accuracy in the second generation of tools including panX [7], PIRATE [8], Panaroo [9], PPanGGOLiN [10], PEPPAN [4], and panTA [3] among others (though it should be noted that constructing pangenomes accurately can be difficult [11] and that these tools are not without their issues and data anomalies [12]). With the increase in research and interest in pangenomic analyses comes an influx in the misuse of the term, with recent calls being made to be clear with the terminology we use [13].

Pangenomics typically separates strain-level diversity between genomes into definitions of ‘open’ and ‘closed,’ where an open pangenome consists of a large number of accessory genes and, thus, more intra-species diversity in comparison with closed pangenomes which contain very little accessory gene content [2]. Although these definitions have been widely adopted, they can be problematic as they lack consistency across studies. For example, Zhou et al*.* [4] points out that the pangenome of *Streptococcus pneumoniae* has been identified as both open [14] and closed [15], and that the calculated rate of pangenome expansion of *Streptococcus* has been reported as 4.4 [4] and 62 [16] genes per novel genome. Further, the estimated number of genes core to *Pseudomonas aeruginosa* has been estimated to be 665 [17], 2,503 [18], and 3,264 [7]. These differences between studies are likely due to methodologic issues such as the number of genomes included, the definition of a core gene (e.g., present in 99% versus 100% of all genomes), variation in the genomic quality filters used, how homologous gene clusters are defined (e.g., percent identity thresholds), and the other assumptions built into different softwares (e.g., whether gene synteny is considered). Of course, this level of variability between studies is not unique to pangenome research, but caution should be used when reporting and comparing pangenomes across the literature.

## The factors that shape the pangenome

Arguably, what drives the diversity observed within pangenomes and whether this diversity is under evolutionary selective pressure [19,20] remains an open question. When considering pangenome evolution, the unit of selection can be defined as the bacterial genome and/or the bacterial gene. Arguments have been made that accessory genes are under adaptive pressures [21] (driven by, for example, shared function [22,23] or niche-associated fitness [24]), negative frequency-dependent selection (e.g., genetic elements such as bacteriocins and antimicrobial resistance genes being more beneficial to the host when they are not abundant in the population due to interstrain competition [25]), selfish elements (e.g., mobile genetic elements such as transposons [26]), or neutral effects [27,28]. It is most likely that different genetic elements within a pangenome experience different pressures and that these can conflict with each other depending on whether we view the unit of selection as the gene or genome [19].

What contributes to the observed diversity of bacterial species? This diversity is often measured by ‘pangenome fluidity’—the average proportion of genes not shared by any two genomes in a species [29,30]. For example, it has been observed that organisms with larger genomes are more likely to have more fluid pangenomes [29]. Similarly, a large effective population size (the number of individuals who contribute to population growth in the next generation [31,32]) has been found to correlate with a large accessory genome [24,27,33]; however, more recently, particular lifestyle traits have been found to be better correlated with pangenome fluidity than effective population size [29]. These lifestyle traits include elements of the bacteria's habitat such as whether it is host-associated or free-living, and elements of how it interacts with its environment such as whether it is motile or not [29]. Habitat has been shown to influence pangenome features [34]. For example, obligate host-associated species have less diverse pangenomes than free-living species [29] and features of the pangenome differ between organisms in soil and aquatic environments [34]. More generally, organisms such as *E. coli* which live in a variety of environments (e.g. soil, animal gut) and can act as opportunistic pathogens have more open pangenomes [35] than range-restricted bacteria, such as symbionts, which have more conserved genomes with less within species diversity [36]. More generally, the environment that a species lives in affects its opportunities and necessities to gain gene novelty, whether by gene duplication and neofunctionalization or interactions with other members of their community via, for example, horizontal gene transfer (HGT).

## The effect of ecology on the pangenome

### Horizontal gene transfer and gene sharing

One of the most obvious ways to affect the genetic content of the pangenome is via gene sharing mechanisms. Rates of HGT are necessarily driven by ecology because HGT requires physical proximity; for example, conjugation requires the donor and recipient cells to be either in direct contact or to connect via a short pilus bridge [37,38]. It follows that bacteria are more likely to undergo HGT if they co-occur in a shared ecological niche [39–41]; further, organisms in a shared niche may experience shared evolutionary pressures and thus benefit from acquiring advantageous genes [42]. Interestingly, it has recently been identified by two independent studies [42,43] that organisms within a niche undergo HGT irrespective of phylogenetic similarity or other environmental factors [42,43]; that is to say, even phylogenetically unrelated organisms that share an environment can influence each other’s pangenome. This is seen in the observed gene variation in *Listeria monocytogenes* isolates collected from soil environments, which was associated with environmental factors (e.g., temperature) but also bacterial community composition including influence from bacteria in different phyla [44].

Rates of HGT within a niche can be influenced by ecological factors [19]. For example, stress responses—including to nutritional stress—can increase rates of HGT (reviewed in [19]); this means that the pangenome of an organism can be affected by the general levels of stress in the niches that they occupy. This could, for example, influence the pangenome of organisms that continually occupy stressful environments such as those associated with low nutrient availability and extreme pH/temperatures. Further, particular elements of the pangenome, such as plasmids encoding antimicrobial resistance genes, might be selected for in such environments, driving selective pressures on the pangenome [45]. Alongside the ability of HGT to bring new genes into a genome, it can also promote novel functions (e.g., by transfer of regulatory elements) [46], having knock-on effects on pangenome structure. Separately, bacterial communication systems such as quorum sensing can affect how bacteria interact with each other. Although initially identified as an intra-species communication tool [47], it was quickly shown that quorum sensing communication could be recognized across bacterial species [48] and that elements (e.g., AI-2) of the quorum sensing machinery are highly conserved across the bacterial tree of life [49]. Now appreciated for its ability to allow inter-species bacterial communication, quorum sensing has also been shown to affect rates of HGT on a micron scale: some quorum sensing systems—some of which regulate conjugation machinery—can alter their range of communication in a way that results in a highly localized regulation of HGT mechanisms such as integrative and conjugative elements and phage [50].

### How intra and interspecies interactions affect a bacteria's pangenome

Strains of a given species that occupy different environmental niches can diversify into ‘ecotypes’—strains of the same species that possess different genetic and phenotypic characteristics across niches [51]. This is due to a gene being beneficial in a particular environment but not in others due to gene-by-environment interactions [20]. Thus, the broader the host/environment range of a bacteria, the more open its pangenome is expected to be. However, despite this broad inter-niche diversity, often large amounts of intra-species diversity can also be maintained within a given niche [52] (sometimes referred to as the ‘local pangenome’ [53]). This diversity can allow the population to react to changes in its environment [52,54]. Some of this diversity can be explained by the self-propagation of selfish elements or the rate of HGT in a population being higher than the rate evolution can act to remove unwanted material by selection [55], but these factors are thought to be small. While our knowledge of why this occurs remains incomplete, we have identified some factors as to how strains and their environment can interact to affect a bacteria's pangenome.

A bacterial population can diversify within an environment. Perhaps the most intuitive example of this is through resource partitioning; starting from a clonal population, *E. coli* can rapidly diversify to fill different nutritional niches [56] and can form stable co-occurring populations [57]. Further, intra-species populations of bacteria can diversify into spatial structures within the niche, which have been shown theoretically and empirically to increase genetic diversity within a population [58,59]. In more complex environments where a particular gene/function is needed by the population but not necessarily by every strain within the community, intra-species bacterial populations can diversify by division of labor, with ‘leaky functions’ (e.g., production of a public good) being lost in some but not all members of the community [20] (the Black Queen hypothesis [60]).

Intra-species diversity within a niche can also be maintained via intra-species competition. Negative frequency-dependent selection can drive intermediate gene frequency of bacteriocins and their immune genes within competing strains of a species [25]. Further, ‘rock–paper–scissors’ dynamics between resistant, toxin-producing, and sensitive strains (where resistant displaces toxin-producing due to a growth rate advantage, toxin-producing kills sensitive, and sensitive outgrows resistant) maintain repertoires of accessory genes via complicated, multi-layer ecological interactions [61].

Inter-species interactions can also affect the pangenome. In addition to the impacts of HGT between organisms living in shared niches (see above), microbes can interact with each other in various ways that affect their pangenome. Microbe pairs can compete with each other to decrease the fitness of one (amensalism) or both (competition) organisms, or they can interact positively to improve the fitness of one (commensalism) or both (mutualism) members, or form an exploitative relationship (fitness of one organism increases while the other decreases) [62,63]. Cross-feeding—the sharing of metabolites as energy between organisms [62,64]—occurs in many, if not all, mixed microbial communities and can be exploitative, commensalistic, or mutualistic [62,64]. Cross-feeding can lead to division of labor within microbial communities, the generation of subcommunities, and highly reduced genomes in mutualistic organisms who lose the ability to generate a shared metabolite themselves [62], all of which are factors that can shape the pangenome. For example, cooperative microbiomes have smaller genomes in general than competitive communities, which have larger genomes and more variability in bacterial ‘weapons’ such as antimicrobial resistance genes [65]. Competitive interactions between organisms can promote negative frequency-dependent selection and thus intermediate frequencies of particular genes. For example, Domingo-Sananes et al. [20] postulates that competition between a bacteria and an infecting phage can lead to accessory genes within the bacteria's pangenome; if a surface receptor is both a receptor for phage and an important nutrient, the pressures from both nutrition and phage attack could result in the responsible gene(s) being at intermediate frequencies in a phage-ridden environment [20].

## Detecting pangenomes directly from shared microbial niches: pangenomes within microbiomes

To study pangenomes, many studies have relied on sequencing singular genomes; to study intra-species variability within a population, often the community is cultured and a small number of individual clones are isolated individually and sequenced (e.g. [66,67]). However, these methods vastly underestimate the pangenomic richness of a species within a community and may not accurately represent the relative abundance of pangenomic variation in the population. To properly understand how ecological interactions shape a bacteria’s pangenome, we need to be able to detect pangenomes *in situ* from within studies of mixed microbial communities. Typically, this means relying on metagenomic DNA sequencing data as a proxy for the pangenomic diversity within a given microbiome. Ever-improving sequencing technologies and bioinformatic software can help us elucidate the pangenomes of microbes within and between mixed microbial communities such that we can better understand how their social lives affect a bacteria’s gene repertoire.

**Figure 2:**
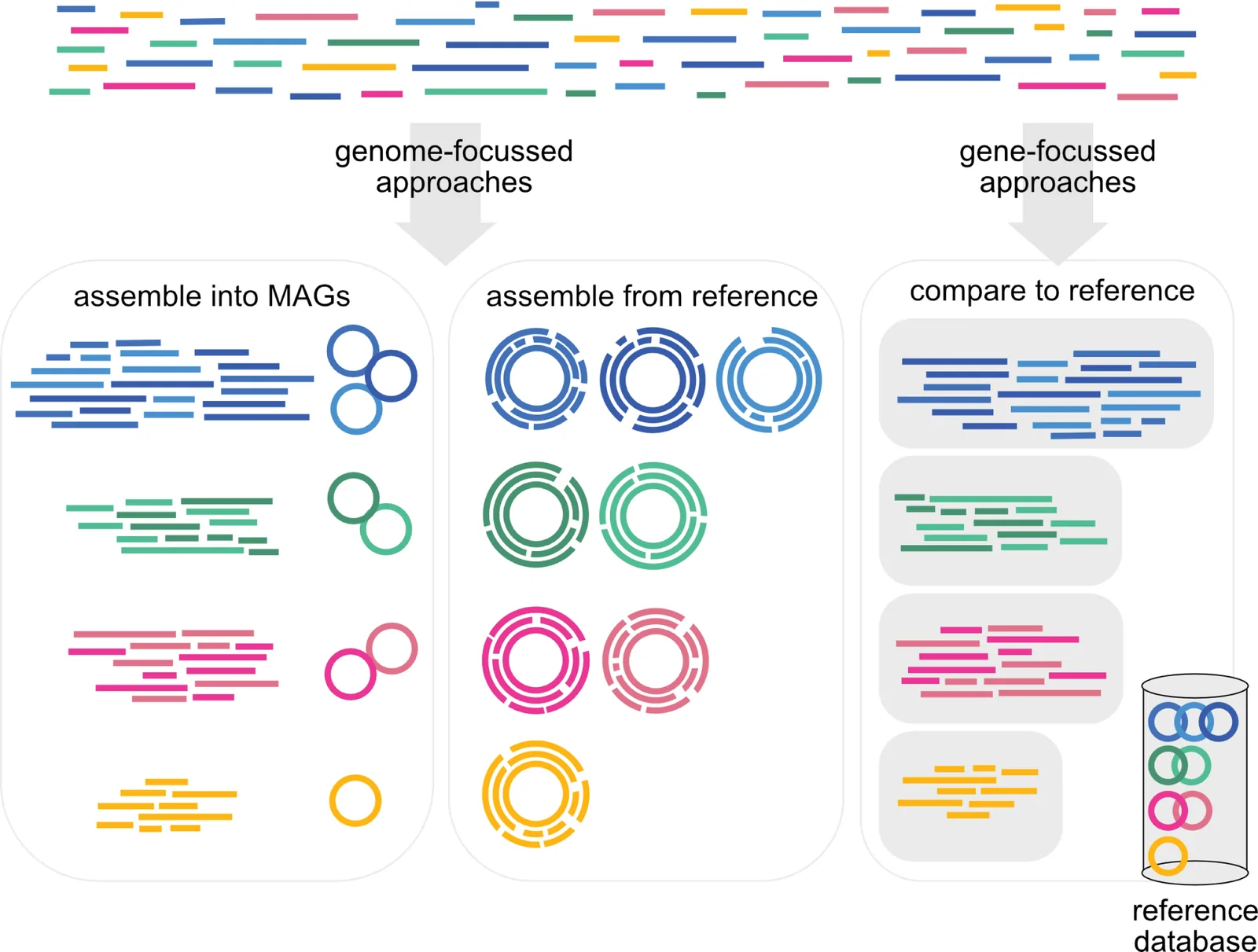
A schematic of how metagenomic sequencing can be used for pan-metagenomic analysis These methods can be split into genome- and gene-focused approaches that result in either metagenomic assembled genomes (MAGs) or gene sets belonging to a given taxon. Genome-focused approaches can be split into *de novo* assembly into MAGs or reference-based assembly; gene-focused approaches involve comparing reads to reference genomes without assembly.

Metagenomics is the use of short- or long-read DNA sequencing of mixed microbial communities in order to identify the totality of their genetic content [68,69]. Although the word ‘strain’ is often used with different meanings, strain- or genome-level metagenomic analysis allows us to identify any differences in gene content observed between strains of the same species within or between different microbial communities; the totality of a gene repertoire belonging to a given taxon from a microbiome or set of microbiomes has been referred to as a ‘meta-pangenome’ [70,71]. Efforts to identify the pangenome of a bacterium from metagenomic data typically fall into two categories: genome- or gene-focused approaches (Figure 2).

### Genome-focused: strain-level identification from metagenomes

Many bioinformatic tools have been developed to identify genomes from whole-genome shotgun metagenomic data. The first of these methods focusses on generating MAGs [72,73] by collecting assembled contigs into bins based on estimates (e.g. GC content, abundance in the sample etc.) that they belong to the same genome [74]. There are also tools, such as DESMAN [75], which are designed to refine MAGs by using the SNP-level diversity within genes core to each MAG to infer the accessory gene content of within-MAG strains. A second approach uses reference (pan)genomes to infer genetic diversity. For example, tools such as PanPhlAn [76] and MIDAS [77] use representative reference genomes to build a pre-computed pangenome reference dataset, then recruit reads from the metagenome to it before identifying strains. A more recent tool, PHLAME [78], takes a slightly different approach by first using a reference genome-based approach (as above) and then identifying novel accessory genes by using a phylogenetic approach based on the assumption that strains share a common ancestor.

However, many of these tools cannot fully resolve strain-level diversity within a metagenome, making it difficult to investigate the full extent of pangenomes within microbiomes. For example, accessory genes are often not able to be placed in MAGs due to their low frequency or recent horizontal transfer event. High-quality MAGs or species-specific gene sets are imperative to the study of pangenomes. MAG generation is imperfect and rarely results in complete genomes without contamination. Instead, MAGs are often composites of multiple genomes, grouping multiple strains (and sometimes species [79]) into the same bin. Further, MAGs often miss accessory genes, which are not at the same relative abundance/frequency as the core genes in the MAG [70]. Simply put, intraspecies diversity necessarily complicates MAG generation [70]. Similarly, relying on reference genome databases is problematic in that any novelty in a bacteria's pangenome will be missed. Tools such as DESMAN—with its accessory-gene-aware refinement of MAGs—and PHLAME—with its sensitivity to novelty—are the potential exceptions when looking for a genome-focused tool to identify a species pangenome from metagenomic sequencing.

### Gene-focused: read recruitment to identify meta-pangenomes

In contrast with genome-focused investigations, we can also ignore the concept of a strain/genome and focus instead on identifying the total catalogue of genes from a particular taxon present in a sample. Typically, gene-focused approaches involve read recruitment from metagenomic sequencing to reference genomes or pangenomes [71] or a focus on differential gene presence/absence patterns across samples or metadata categories [80]. These methods have an advantage over genome-focused approaches in that they do not rely on resolving genomes from metagenomes. This means that these methods can result in higher resolution detection of important accessory genes; for example, Shekarriz et al. successfully identified accessory genes which engrafted during faecal microbiota transplant (FMT) where they could not be identified at the strain- or species-level [80]. However, like the use of reference databases for genome-focused approaches, relying on reference databases is disadvantageous in that it can only identify genes, which have been previously identified as part of the taxon in the reference (pan)genome. Further, gene-focused methods are unable to differentiate core versus accessory genes, and genes that are horizontally transferred may not be able to be recruited to a single reference.

### The current state of meta-pangenomes

Although these investigations are arguably in their early days, a few studies have pioneered the study of pangenomes within microbiomes. Most of these studies focus on less complex communities—such as the vagina [81] or skin [82]—where MAGs are easier to compute and/or use long-read sequencing [83] to improve MAG quality. Studies have correlated the presence/absence patterns of accessory gene content with particular ecological attributes. Delmont et al. identified accessory genes within *Prochlorococcus* that correlated with whether they lived in high- or low-light environments [71]. Minich et al. used long-read sequencing to form complete MAGs and associate particular accessory genes within a species to metadata categories including linear growth and stunting in the gut microbiomes of a cohort of children at risk of undernutrition [83]. Other studies have used pangenome diversity to better understand evolutionary pressures and ecological assortment within microbiomes. For example, Baker et al. used estimates of relatedness of strains within a species’ pangenome to determine that closely related strains of *Streptococcus epidermidis* and *Cutibacterium acnes* on the skin coexist with each other under a neutral model of evolution [82]. Further, Madi et al. show that the species richness of the microbial community predicts the intra-species genetic diversity [84], but that this relationship breaks down at very high levels of diversity. These findings are consistent with the diversity-begets-diversity hypothesis, as communities assemble but eventually ecological constraints limit pangenome diversity [84]. Fewer studies have used meta-pangenomics to study the effect of inter-species microbial interactions on pangenome dynamics; in the context of FMT, the ability of a new strain to replace an existing one in the gut microbiome has been shown to be influenced by the presence of other bacterial species within the donor and recipient communities [85] suggesting the effect of one organism's pangenome on another's. Collectively, meta-pangenomics has been used to understand how a taxon's pangenome is shaped by ecological factors.

## Perspectives on the future

Our understanding of how living socially in mixed communities affects a prokaryote's pangenome will only continue to evolve alongside sequencing technologies, bioinformatic methods, and innovative *in vitro* studies. The detection of pangenomes directly from metagenomic sequencing will become easier and more complete with long-read sequencing, new bioinformatic methods which can scale with the growth of genome databases [86], and other future innovations, potentially paving the way for pangenome graphs to replace reference genomes for some applications (as has been adopted in some eukaryotic research [87]). Meta-pangenomics is a powerful tool to study microbial ecology; for example, meta-pangenomics could be helpful in untangling the co-occurrence dynamics between intra-species strains and in understanding how microbe-microbe interactions influence microbial diversity. Other methods not reviewed here—such as single cell isolation [88] and sequencing and culture enriched metagenomics [79,89]—as well as combinations of methods (e.g., genomics and metagenomics [90]) also have the potential to innovate in this field.

## Summary

- A bacteria’s pangenome is shaped by the other organisms they interact with and the environmental niches they occupy.
- Here, I review available bioinformatic tools able to detect strain-level bacterial genomes from metagenomic sequencing data.
- I explore meta-pangenomics and how this concept is being used to study the effect of social interaction on the genetic diversity of bacteria within mixed microbial communities.

## Open Access

Open access for this article was enabled by the participation of University of Manchester in an all-inclusive Read & Publish agreement with Portland Press and the Biochemical Society under an agreement with JISC.

## Abbreviations

FMT: faecal microbiota transplant
HGT: horizontal gene transfer
MAGs: metagenomic assembled genomes
